# Performance of single-agent and multi-agent language models in Spanish language medical competency exams

**DOI:** 10.1186/s12909-025-07250-3

**Published:** 2025-05-07

**Authors:** Fernando R. Altermatt, Andres Neyem, Nicolas Sumonte, Marcelo Mendoza, Ignacio Villagran, Hector J. Lacassie

**Affiliations:** 1 Division of Anesthesiology, School of Medicine, Pontificia Universidad Católica de Chile, Marcoleta 377, 8320000 Santiago, RM Chile; 2 Department of Computer Science, School of Engineering, Pontificia Universidad Católica de Chile, Vicuña Mackenna 6840, 7820436 Santiago, RM Chile; 3 Department of Kinesiology, Health Sciences School, Pontificia Universidad Católica de Chile, Vicuña Mackenna 6840, 7820436 Santiago, RM Chile; 4 National Center for Artificial Intelligence (CENIA), National Research and Development Agency (ANID), Vicuña Mackenna 6840, 7820436 Santiago, RM Chile; 5 Millennium Institute for Foundational Research On Data (IMFD), Pontificia Universidad Católica de Chile, Vicuña Mackenna 6840, 7820436 Santiago, RM Chile

**Keywords:** Large language models, Medical decision-making, Spanish medical contexts, Medical AI., GPT-4o

## Abstract

**Background:**

Large language models (LLMs) like GPT-4o have shown promise in advancing medical decision-making and education. However, their performance in Spanish-language medical contexts remains underexplored. This study evaluates the effectiveness of single-agent and multi-agent strategies in answering questions from the EUNACOM, a standardized medical licensure exam in Chile, across 21 medical specialties.

**Methods:**

GPT-4o was tested on 1,062 multiple-choice questions from publicly available EUNACOM preparation materials. Single-agent strategies included Zero-Shot, Few-Shot, Chain-of-Thought (CoT), Self-Reflection, and MED-PROMPT, while multi-agent strategies involved Voting, Weighted Voting, Borda Count, MEDAGENTS, and MDAGENTS. Each strategy was tested under three temperature settings (0.3, 0.6, 1.2). Performance was assessed by accuracy, and statistical analyses, including Kruskal–Wallis and Mann–Whitney U tests, were performed. Computational resource utilization, such as API calls and execution time, was also analyzed.

**Results:**

MDAGENTS achieved the highest accuracy with a mean score of 89.97% (SD = 0.56%), outperforming all other strategies (*p* < 0*.*001). MEDAGENTS followed with a mean score of 87.99% (SD = 0.49%), and the CoT with Few-Shot strategy scored 87.67% (SD = 0.12%). Temperature settings did not significantly affect performance (*F*2*,*54 = 1*.*45, *p* = 0*.*24). Specialty-level analysis showed the highest accuracies in Psychiatry (95.51%), Neurology (95.49%), and Surgery (95.38%), while lower accuracies were observed in Neonatology (77.54%), Otolaryngology (76.64%), and Urology/Nephrology (76.59%). Notably, several exam questions were correctly answered using simpler single-agent strategies without employing complex reasoning or collaboration frameworks.

**Conclusions and relevance:**

Multi-agent strategies, particularly MDAGENTS, significantly enhance GPT-4o’s performance on Spanish-language medical exams, leveraging collaboration to improve diagnostic accuracy. However, simpler single-agent strategies are sufficient to address many questions, high-lighting that only a fraction of standardized medical exams require sophisticated reasoning or multi-agent interaction. These findings suggest potential for LLMs as efficient and scalable tools in Spanish-speaking healthcare, though computational optimization remains a key area for future research.

**Supplementary Information:**

The online version contains supplementary material available at 10.1186/s12909-025-07250-3.

## Background

The rapid evolution of large language models (LLMs) has revolutionized the field of artificial intelligence, unlocking capabilities with broad applications across numerous disciplines [1–3]. In the medical domain, LLMs have demonstrated significant promise in advancing medical decision-making (MDM) by enhancing diagnostic accuracy, personalizing therapeutic strategies, and optimizing resource utilization [4–9]. For example, GPT-based systems have been employed to generate clinical summaries, assist with complex diagnoses, and predict patient outcomes from large datasets, fundamentally transforming patient care approaches [10–12]. In the context of medical education, [13] assessed the performance of ChatGPT versions 3.5, 4, and 4 V on the EUNACOM, a key national medical licensing exam in Chile. These evaluations demonstrated ChatGPT’s ability to pass the exam, albeit with varying proficiency level across medical disciplines and model iterations. However, these results also underscored significant limitations, such as biases in AI training data and challenges in linguistic adaptability [13]. Language barriers, in particular, remain a critical obstacle in applying LLMs to non-English contexts [14–16].

Spanish, the second most spoken native language globally, represents a vast demographic that could greatly benefit from innovations in AI-driven healthcare [17]. Yet, current LLMs, predominantly trained on English-language datasets, face substantial linguistic and cultural limitations when applied to Spanish medical contexts. For instance, studies like that of Guillen-Grima et al. [18] show that GPT-4, despite achieving high scores on Spain’s Medical Residency Examination (MIR), struggled with domain-specific nuances and multimodal questions. Similarly, Vera [19] highlights the importance of linguistic concordance in clinical communication, emphasizing the need for AI systems that account for the complexities of Spanish medical terminology to improve patient-centered care. These findings point to the need for robust adaptations of LLMs tailored to Spanish-speaking populations.

The architecture and configuration of LLMs also play a critical role in their performance on high-stakes medical tasks. Single-agent frameworks, such as GPT-4o, leverage advanced techniques like Chain-of-Thought (CoT) reasoning [20] and self-reflection [21–26], and have shown commendable performance in complex medical exams such as the USMLE. However, single-agent models often falter when tasked with interdisciplinary cases that demand collaborative reasoning [27, 28]. In contrast, multiagent frameworks, exemplified by MEDAGENTS [29] and MDAGENTS [30], integrate diverse perspectives through iterative discussions and voting mechanisms among specialized agents. These collaborative configurations mimic real-world medical decision-making processes, enhancing robustness and accuracy, particularly in error-prone scenarios.

In this context, our study addresses a critical gap in the evaluation of LLMs for Spanish-language medical competency exams. Specifically, we examine the performance of GPT-4o on the National Single Examination of Medical Knowledge (EUNACOM), a standardized assessment essential for medical licensure in Chile. This exam presents a unique challenge as it evaluates specialized medical knowledge in Spanish, offering a rigorous testbed for assessing the efficacy of LLMs in non-English healthcare contexts.

Through a comparative analysis of single-agent and multiagent configurations, we investigate key performance metrics, including accuracy, response time, and computational efficiency, as measured by API calls. Advanced prompting techniques, such as CoT and MEDPROMPT, are employed for single-agent models, while collaborative frameworks are used to evaluate multiagent systems. This approach allows us to elucidate the strengths and limitations of these configurations and to identify strategies that enhance the adaptability of LLMs in Spanish-speaking medical scenarios. By contributing to the broader discourse on adapting AI systems for diverse linguistic and cultural contexts, this research seeks to advance the integration of LLMs into global healthcare.

Ultimately, this study bridges a critical gap in the understanding of LLM applications in Spanish medical education and lays the groundwork for future innovations in multilingual AI-driven healthcare. By addressing the linguistic and methodological challenges inherent in adapting LLMs for Spanish-speaking populations, we aim to catalyze the development of inclusive and effective AI tools that align with the needs of diverse healthcare systems worldwide.

## Methods

In this study, we investigated the performance of GPT-4o on the National Single Examination of Medical Knowledge (EUNACOM), a stringent medical licensure assessment administered in Chile. We evaluated the model’s single-agent and multiagent configurations and examined how different prompting strategies and collaborative framework influenced accuracy, consistency, adaptability, and response times. Additionally, we assessed the impact of various temperature settings to understand how response variability and uncertainty affect performance in a Spanish-language, domain-specific medical context.

### EUNACOM as a Benchmark for medical competency

The EUNACOM is a high-stakes, standardized test designed to validate the medical knowledge of physicians practicing in Chile. It encompasses a broad range of multiple-choice questions (MCQs) covering internal medicine, surgery, pediatrics, obstetrics and gynecology, and other core medical domains. Its comprehensive scope and complexity make it an ideal benchmark for evaluating the capabilities of large language models in specialized, context-rich medical scenarios.

For this study, we curated a dataset of 1,062 publicly available and previously administered EUNACOM questions. All items underwent preprocessing, including orthographic standardization and exclusion of any image-based or multimodal content, to ensure compatibility and consistency. Answer choices were randomized for each run to minimize ordering biases and enhance robustness. Each question in the dataset included a predefined correct answer that served as the evaluation key. The assessment is based on multiple-choice questions (MCQs); therefore, no metrics are used to compare open-ended text responses. It is a multiple-choice format The evaluation process was fully automated, comparing model-generated answers against these established correct answers. This careful curation and automated assessment methodology ensured that the resulting dataset provided a balanced, unbiased platform for model evaluation.

### Experimental design

We evaluated GPT-4o under two primary configurations: a single-agent setting and a multiagent framework. Each configuration was tested across three temperature conditions (0.3, 0.6, and 1.3) to capture the effects of varying response diversity and uncertainty on performance. The chosen temperature values reflect a range of response behaviors, from deterministic outputs to more exploratory and diverse responses. At the lower end, a temperature of 0.3 minimizes randomness, encouraging highly deterministic and precise outputs that are critical for high-stakes tasks like medical licensure exams. This setting evaluates the model’s intrinsic confidence and consistency. A medium temperature setting of 0.6 introduces a balance between focused reasoning and some degree of exploration, simulating real-world scenarios where nuanced reasoning or multiple valid approaches may be applicable. Finally, a higher temperature of 1.3 maximizes diversity in responses, fostering exploration and adaptability, which is particularly useful for understanding the model’s robustness in handling ambiguous or unconventional questions. By using this range of temperatures, we aimed to assess the model’s stability and adaptability under varying degrees of response variability.

Each configuration was tested twice at each temperature setting to ensure robust and reliable results. These experiments provided a comprehensive evaluation of the model’s capacity to adapt its reasoning processes across deterministic and stochastic conditions.

### Single-agent configuration

In the single-agent setup, we explored various prompting techniques to assess the model’s capacity to handle specialized medical knowledge. Zero-shot prompting was used to present each question without additional context, relying solely on the model’s pretrained knowledge [31]. Few-shot prompting involved providing a small set of example question–answer pairs before each query, which refined the model’s reasoning within the specific domain [32]. Advanced techniques incorporated chain-of-thought (CoT) reasoning [20], prompting the model to explicitly outline its reasoning steps. This approach was further coupled with self-reflection [21], allowing the model to review and refine its responses. Additionally, combinations of CoT with few-shot examples and the MEDPROMPT [33] method, designed specifically for medical contexts, were evaluated.

All single-agent strategies were tested across the three temperature settings, enabling a detailed analysis of the effects of controlled variability on accuracy and reliability. A comprehensive description of these techniques is provided in Appendix A.

### Multiagent configuration

The multiagent framework emulated a collaborative diagnostic environment, featuring multiple instances of GPT-4o working in concert. The strategies included simple voting, where six independent agents provided responses, and the majority vote determined the final answer. Weighted voting incorporated confidence-based weighting schemes, such as the Borda count [34], to reflect agent certainty in the consensus. Role-specific frameworks, such as MEDAGENTS, assigned agents domain-specific roles (e.g., cardiologist, pediatrician), mimicking interdisciplinary collaboration in clinical practice [29]. The MDAGENTS configuration further adapted agent reasoning strategies to the diagnostic or therapeutic context, enhancing the relevance and accuracy of responses [30].

Each multiagent strategy was tested across the same temperature settings to directly compare their performance with single-agent configurations. Detailed descriptions of the frameworks, including voting mechanisms and agent roles, are provided in Appendix B.

### Language model and implementation

All experiments employed OpenAI’s GPT-4o, a state-of-the-art multilingual language model with demonstrated proficiency in Spanish. We used the base version without additional fine-tuning to provide an out-of-the-box evaluation. The responses were limited to 3,048 tokens to ensure completeness while avoiding excessive verbosity. Experiments were conducted on a cloud-based platform via Python 3.8 and the langchain library, leveraging the OpenAI API to facilitate reproducibility and scalability.

### Evaluation metrics and statistical analyses

We performed all analyses on the dataset of 1,062 EUNACOM questions (*n* = 1*,* 062). Accuracy, defined as the proportion of correctly answered questions, was the primary outcome measure, reported as the mean ± standard deviation (s.d.). Additionally, we recorded the average number of API calls and the mean response time per query to assess computational resources and execution speed.

The normality of the data distribution was tested using the Shapiro–Wilk test, which indicated that the data were not normally distributed (*p* < 0*.*05). As a result, nonparametric statistical tests were employed. The Kruskal–Wallis test was used to compare accuracy across strategies and conditions, with an alpha level (*α*) set at 0.05. Post hoc pairwise comparisons were conducted using the Mann–Whitney U test, with the Benjamini–Hochberg procedure applied to correct for multiple comparisons.

All statistical analyses were performed in R (version 4.2.2) using open-source packages. The statistical codes, subsets of the data, and detailed p values are provided in Appendix C.

### Ethical and regulatory considerations

This work adhered to established ethical guidelines governing standardized examination materials. All EUNACOM items were anonymized, and the dataset did not contain any patient-identifying information. Since this research did not involve human participants, institutional ethical review was not needed.

### Reproducibility

To ensure robust and reproducible results, all the experimental conditions were standardized, and each configuration was repeated twice at each temperature. Appendices A and B provide comprehensive documentation of prompts and configurations, enabling independent verification and replication of the findings.

## Results

### Overall performance

**Fig. 1:**
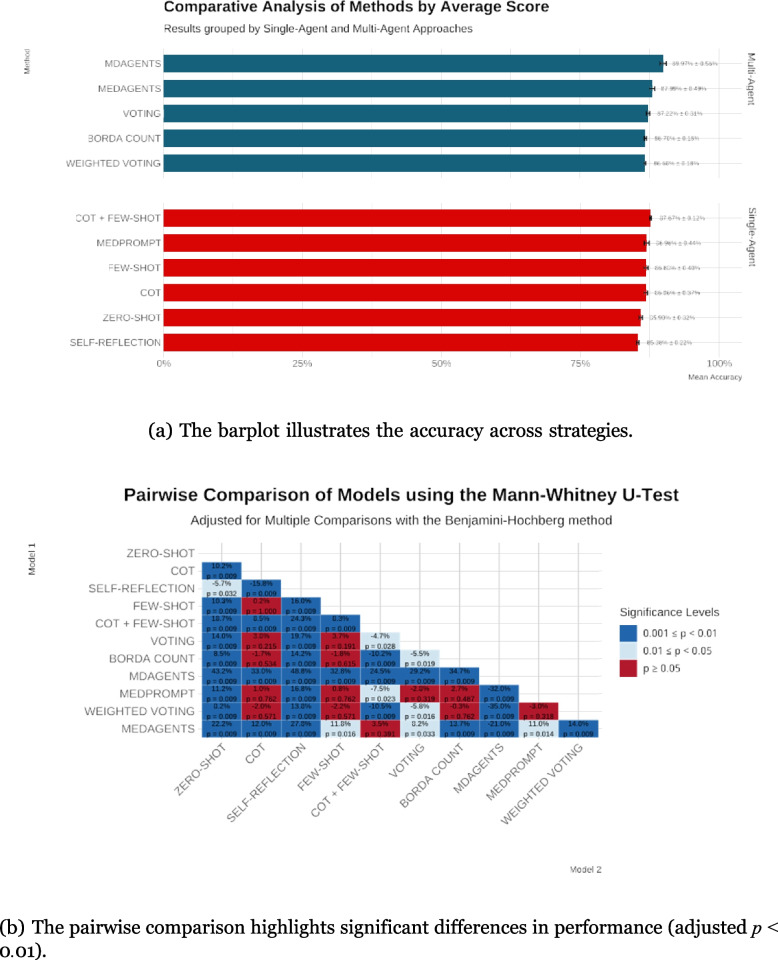
Performance metrics for all evaluated strategies on the EUNACOM exam

Our evaluation of GPT-4o on the EUNACOM examination revealed substantial differences in performance across the tested strategies and configurations (Table 1). Multiagent frameworks consistently outperform single-agent methods, with MDAGENTS emerging as the top performer, as illustrated in Figure 1a. MDAGENTS achieved a mean accuracy of 89.97% (SD = 0.56%), significantly surpassing simpler approaches, including zero-shot (85.90%, SD = 0.32%) and self-reflection (85.38%, SD = 0.22%) (all adjusted *p* < 0*.*01).

**Table 1:** Performance metrics for all evaluated strategies on the EUNACOM Exam. Mean scores, standard deviations (SD), API calls, and mean completion time (in sec- onds) are shown

Category	Strategy	Accuracy (Mean % ± SD)	API Calls	Time (s)
Single-agent	COT + Few-Shot Few-Shot	87.67% ± 0.12% 86.88% ± 0.40%	1.00 1.00	1.74 1.61
	CoT MEDPROMPT	86.86% ± 0.37%	1.00	2.26
		86.96% ± 0.44%	1.00	2.95
	SELF-REFLECTION	85.38% ± 0.22%	2.65	4.15
	ZERO-SHOT	85.90% ± 0.32%	1.00	1.53
	MDAGENTS	89.97% ± 0.56%	21.14	192.44
	MEDAGENTS	87.99% ± 0.49%	17.00	63.95
Multi-agent	VOTING	87.22% ± 0.31%	6.00	12.51
	BORDA COUNT	86.70% ± 0.18%	6.00	13.03
	Weighted Voting	86.68% ± 0.18%	6.00	12.43

Single-agent approaches that incorporate prompt engineering and guided reasoning—such as few-shot (86.88%, SD = 0.40%), MEDPROMPT (86.96%, SD = 0.44%), and CoT (86.86%, SD = 0.37%)—consistently outperform zero-shot and self-reflection (all adjusted *p* < 0*.*01 for key comparisons). Notably, the CoT + few-shot configuration achieved exceptional consistency (SD = 0.12%), highlighting the benefits of structured reasoning strategies.

### Pairwise comparisons and statistical significance

Pairwise comparisons confirmed that advanced reasoning and multiagent approaches yield substantial benefits over baseline techniques, as shown in Figure 1b. For example, MDAGENTS outperforms zero-shot by an average margin of 4.06 percentage points (adjusted *p* < 0*.*01) and self-reflection by 4.60 percentage points (adjusted *p* < 0*.*01). CoT + few-shot, MEDAGENTS, and other enhanced methods also achieved statistically significant improvements, further emphasizing the value of structured prompting and collaborative reasoning.

### Robustness to temperature variation

**Fig. 2:**
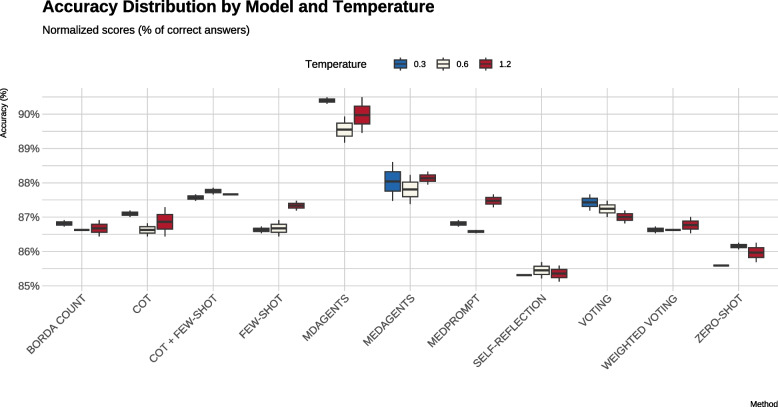
Boxplot of strategy performance across varying temperature settings. Stability across all configurations highlights the robustness of the methods

Neither the single-agent nor the multiagent strategies showed significant sensitivity to temperature adjustments (0.3, 0.6, 1.3), as evidenced by the Kruskal–Wallis test results (*p* > 0*.*05 for all comparisons). This stability underscores the robustness of the observed performance improvements, demonstrating their resilience to variations in sampling stochasticity and enhancing their reliability across diverse operational contexts. The performance distributions under different temperature settings are visualized in Figure 2. Detailed statistical results, including pairwise comparisons and Kruskal–Wallis tests for configurations, are provided in Appendix C.

### Computational considerations and consistency

**Fig. 3:**
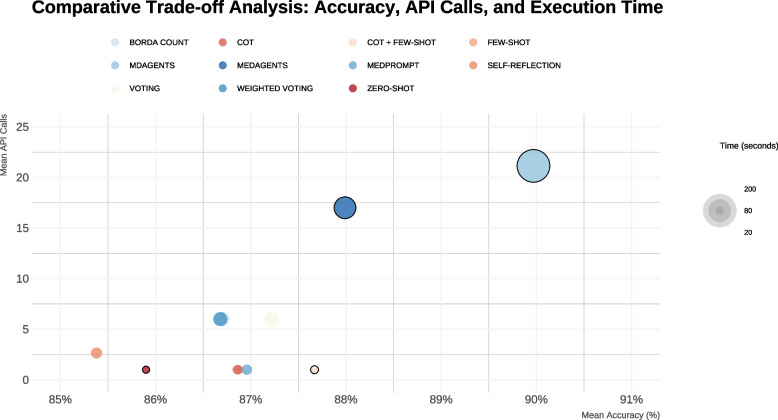
Scatter plot of mean accuracy versus computational requirements (API calls) for all evaluated strategies

While multi-agent strategies offer superior accuracy, they require greater computational resources. In contrast to single-agent methods such as Zero-Shot and CoT, which require only a single API call and complete queries within seconds, MDAGENTS averages 21.14 API calls and approximately 192 seconds per experiment. CoT + few-shot combined strong accuracy (87.67%, SD = 0.12%) with minimal computational overhead, representing an optimal balance between performance and efficiency. Figure 3 illustrates all configurations’ trade-offs between accuracy and computational requirements.

### Performance by topic

A detailed analysis of performance by medical specialty was conducted to assess how each strategy performed across different medical domains. Table 2 presents the average accuracy of all the strategies in each medical area. The results indicate that the highest accuracies were achieved in specialties such as psychiatry (95.51%), neurology (95.49%), and surgery (95.38%). The accuracies of specialties such as neonatology (77.54%), otolaryngology (76.64%), and urology and nephrology (76.59%) were lower. These variations suggest that the model performs better in certain medical domains, possibly because of the complexity of the subject matter or the availability of training data in those areas. Understanding performance by topic is crucial for identifying the strengths and limitations of the model in various medical contexts, as it can guide targeted improvements in areas where the model underperforms. Appendix D provides further details on the accuracy distributions by specialty.

**Table 2:** Average accuracy by medical specialty

Specialty	Average Accuracy (%)
Cardiology	87.73
Surgery	95.38
Dermatology	92.00
Endocrinology	86.97
Gastroenterology	92.39
Gynecology	88.61
Hematology and Oncology	86.29
Infectious Diseases	87.20
Nephrology	87.65
Neonatology	77.54
Neurology	95.49
Obstetrics	86.89
Ophthalmology	82.23
Otolaryngology	76.64
Pediatrics	86.52
Psychiatry	95.51
Respiratory Medicine	80.80
Rheumatology	85.23
Public Health	80.66
Traumatology	83.36
Urology	88.17
Urology and Nephrology	76.59

### Key insights

These findings highlight the substantial benefits of collaborative, multiagent frameworks such as MDAGENTS, which leverage distributed reasoning and specialized roles to achieve superior accuracy in complex medical assessments. Moreover, strategies such as the CoT + few-shot configuration demonstrate that carefully crafted prompts can balance high reliability with minimal computational overhead. Although performance across specialties generally remains strong, certain domains continue to pose challenges, suggesting that further refinements may be necessary to ensure consistently high accuracy in all medical areas. Moreover, the demonstrated robustness to temperature variations and the availability of efficient yet effective approaches underscore the practical potential of these large language models in real-world educational and clinical contexts.

## Discussion

This study underscores the growing potential of large language models (LLMs), specifically GPT-4o, to support medical competency evaluations in Spanish-language contexts. By benchmarking the performance of both single-agent and multiagent configurations on the EUNACOM—a high-stakes licensure exam—we demonstrate that LLMs can serve as scalable, accurate tools for educational and diagnostic tasks. Each strategy presents distinct advantages depending on task complexity, resource constraints, and application setting.

### Performance insights and implications

Our results show that multiagent frameworks—particularly MDAGENTS—significantly outperform single-agent strategies, achieving the highest mean accuracy (89.97%, SD = 0.56%). These findings support prior literature emphasizing the advantages of distributed reasoning in AI systems, where collaboration among specialized agents enhances interpretive depth and robustness [30]. By simulating interdisciplinary clinical reasoning, multiagent frameworks mirror real-world medical decision-making and offer superior performance in complex cases.

Nonetheless, certain single-agent approaches demonstrated competitive accuracy with substantially lower computational costs. The CoT + few-shot configuration, for instance, reached 87.67% accuracy (SD = 0.12%) with minimal API usage, offering a practical alternative for scenarios with limited computational infrastructure. Importantly, many exam questions were answered correctly using simpler prompting techniques, suggesting that complex reasoning is not universally required across the EUNACOM. This highlights an opportunity to selectively apply advanced strategies where they are most impactful—reserving collaborative methods for more ambiguous or interdisciplinary cases.

### Robustness and generalizability

The consistency of performance across different temperature settings (0.3, 0.6, 1.3) reinforces the robustness of both single- and multiagent strategies. The lack of significant performance fluctuations suggests that GPT-4o can maintain accuracy under variable stochastic conditions—a crucial feature for high-stakes, real-world applications.

However, performance varied across medical specialties. High accuracies in psychiatry, neurology, and surgery suggest these domains are well-represented in the model’s training data or better suited to LLM reasoning. Conversely, lower performance in neonatology, otolaryngology, and urology may reflect domain complexity, limited data exposure, or nuanced terminology. These discrepancies highlight areas for targeted model refinement or domain-specific tuning to ensure equitable performance across all specialties.

### Computational trade-offs and practical applications

While multiagent strategies yielded the best results, their implementation comes at a computational cost. MDAGENTS required an average of 21.14 API calls and 192 seconds per experiment, compared to just one call for Zero-Shot or CoT strategies. These resource demands may limit scalability, particularly in low-resource settings or real-time applications.

Given this trade-off, selecting an appropriate configuration should be guided by use-case constraints. In high-stakes clinical simulations or policy development, multiagent frameworks may be justified. In contrast, for medical education or exam preparation, high-performing single-agent strategies (e.g., CoT + few-shot) may offer a more efficient alternative without compromising accuracy. This flexibility enables institutions to match LLM deployment to infrastructure capabilities and pedagogical goals.

### Educational applications

LLMs offer transformative opportunities for medical education, particularly in Spanish-speaking regions. GPT-4o and similar models can serve as interactive tutors, facilitating case-based learning and immediate feedback—a pedagogical approach shown to improve clinical reasoning skills [35, 36]. Beyond tutoring, LLMs can assist in generating high-quality multiple-choice questions and answer rationales, supporting scalable and standardized assessment development [37].

Our research validates these educational applications through GPT-4o’s strong performance on the EUNACOM examination. The differential accuracy across specialties identifies domains where these tools could be most effectively implemented in Spanish-language curricula, while also highlighting areas requiring additional educator guidance.

As Li et al. [38] emphasize, these technologies are redefining medical educator roles from conventional knowledge transmitters to learning navigators who guide critical thinking and information evaluation. This transition is especially valuable in Spanish speaking contexts, where LLMs can help address disparities in clinical educational resources through virtual clinical scenarios and personalized learning paths [38].

Our comparison of prompting strategies offers practical implementation pathways for educators. The strong performance of computationally efficient approaches suggests that even institutions with limited resources can effectively implement LLM-assisted education—a critical consideration for many Spanish-speaking medical schools.

Adaptive learning environments, where AI-generated content dynamically responds to learner progress, can personalize education in ways not previously possible. This is especially valuable in resource-limited settings, where faculty shortages and grading inconsistencies may hinder quality instruction. By demonstrating reliable performance on a standardized Spanish-language exam, our study contributes to the validation of LLMs in such educational roles.

Future research should explore the longitudinal impact of LLM-based tools on medical training, knowledge retention, and clinical performance in Spanish-speaking healthcare systems, as well as their integration into formal curricula.

### Language considerations in Spanish medical contexts

We acknowledge that regional variations exist within the Spanish language, including differences in vocabulary, idioms, and local expressions. However, in the context of standardized medical examinations such as EUNACOM, the language used tends to be linguistically neutral. This neutrality is a characteristic feature of medical discourse, resulting from shared international curricula, scientific literature, and the technical nature of medical terminology, which largely transcends regional dialectical variations.

While the specific impact of Spanish dialectical variations on medical examination performance has not been extensively studied, related research in other languages suggests minimal effects. A randomized controlled trial by Kozato et. al [39]demonstrated that non-native English accents had no statistically significant effect on examiners’ scores in Objective Structured Clinical Examinations (OSCEs). As noted in their findings, *examiners were not biased either positively or negatively towards [non-native English accents] when providing checklist or global scores* [39]. Although this study was conducted in English, it suggests that in standardized medical assessment contexts, linguistic variations may not significantly impact performance outcomes.

Nevertheless, we recognize that in broader healthcare delivery contexts, particularly in direct patient care settings, linguistic variations can be highly relevant. A recent review [40] highlights that the use of local languages in healthcare delivery improves metrics such as patient satisfaction, compliance with medical instructions, and health improvement. This underscores the importance of considering dialectical variations in clinical practice, even if their impact on standardized examinations may be limited.

The version of Spanish used in this study aligns with what is typically understood as Latin American Spanish—common across much of Central and South America, including regions such as Mexico and the Caribbean. Given the standardized nature of the EUNACOM exam, we believe the LLM’s ability to generalize across Spanish-speaking regions is likely to remain robust, especially in formal, medical contexts. It should be noted that dialectal variation, even within standardized Spanish, constitutes a limitation of the study. This is particularly relevant given that we do not currently have access to standardized exams from other Spanish-speaking countries to conduct comparative analyses.

Future work may explore this question more directly by evaluating LLMs across distinct Spanish dialects and clinical terminologies used in different Spanish-speaking countries. Such analyses would further enhance the adaptability and inclusiveness of AI models in global health education and practice, particularly as they extend beyond standardized examinations to clinical applications.

## Conclusion

This study demonstrates the capability of GPT-4o in tackling the challenges of Spanish-language medical licensing examinations, specifically the EUNACOM. By evaluating single-agent and multiagent configurations, we provide valuable insights into the trade-offs between accuracy, computational efficiency, and robustness.

Multiagent frameworks, particularly MDAGENTS, emerged as the most accurate strategy, showcasing the advantages of collaborative reasoning and specialized roles. However, single-agent configurations like CoT + few-shot provide a compelling alternative, delivering high accuracy with minimal computational overhead. These findings highlight the potential of LLMs to enhance medical education and assessment while emphasizing the need for optimizing models for practical and scalable applications.

Future research should focus on addressing the identified gaps in performance across medical specialties and further refining multiagent frameworks to reduce the computational demands. Additionally, expanding the training datasets to better represent Spanish-language medical contexts will be crucial for improving the models’ linguistic and cultural adaptability.

Ultimately, this study contributes to the broader discourse on leveraging LLMs for multilingual healthcare applications, advocating for inclusive and context-sensitive AI systems that can benefit diverse populations globally.

## Supplementary Information

Supplementary Material 1. Our article includes an appendix section as supplementary material for additional context.

## Data Availability

The datasets generated and/or analyzed during this study are partially available. Experimental protocols, including detailed prompting strategies and configurations for both single-agent and multiagent setups, are included in the Appendix. The raw performance data (accuracy scores, API call logs, execution times) and GPT-4 interaction transcripts are available from the corresponding author upon reasonable request. Access to these data requires a formal request outlining the intended use and adherence to data usage terms. The statistical analysis results and the corresponding results are provided in the appendix. This study did not involve human subjects or clinical trials; therefore, trial registration is not applicable.
