# Supplementary material for: Performance of single-agent and multi-agent language models in Spanish language medical competency exams
Source: BMC Med Educ. 2025 May 7;25:666. doi: 10.1186/s12909-025-07250-3 (PMC12057199; doi:10.1186/s12909-025-07250-3)
Supplement: Supplementary file 1 — Supplementary Material 1. Our article includes an appendix section as supplementary material for additional context. [file 12909_2025_7250_MOESM1_ESM.pdf]

Appendix A Prompt Templates used in Single-Agent Methods

This appendix details the prompt templates used for different configurations in our study. Each subsection briefly explains the purpose of the template before presenting it.

A.1 Zero-Shot

This configuration provides no prior examples and relies solely on the model's medical knowledge to answer the multiple-choice question directly.

```
Act as a medical expert answering multiple choice questions.
Select the best answer based on your medical knowledge.
Question: {{question}}
{{answer_options}}
Answer:
```

A.2 Few-Shot

This configuration includes previous examples to guide the model's reasoning, offering context and demonstrating the desired format of the answer.

```
The following are multiple choice questions (with answers) about medical knowledge.
[Example 1]
Question: A 65-year-old man with a history of diabetes, hypertension, smoking, and
dyslipidemia presents with right nasal obstruction persisting for 6 months,
associated with serosanguineous rhinorrhea. Physical examination reveals no
significant findings. What is the most likely diagnosis?
A) Allergic rhinitis
B) Vasomotor rhinitis
C) Nasal polyps
D) Rhinofibroangioma
E) Nasal cancer
Answer: E) Nasal cancer is the most likely diagnosis considering the patients risk
    factors and the clinical presentation.

[Additional examples follow the same format]
Question: {{question}}
{{answer_options}}
Answer:
```

A.3 Chain-of-Thought (CoT)

This configuration encourages step-by-step reasoning, guiding the model to detail its thought process before selecting the final answer.

```
The following are multiple choice questions (with answers) about medical knowledge.
Analyze each case step by step to arrive at the correct answer.
[Example with reasoning]
Question: A 65-year-old man with a history of diabetes, hypertension, smoking, and
dyslipidemia presents with right nasal obstruction persisting for 6 months,
associated with serosanguineous rhinorrhea. Physical examination reveals no
significant findings. What is the most likely diagnosis?
A) Allergic rhinitis
B) Vasomotor rhinitis
C) Nasal polyps
D) Rhinofibroangioma
```

```
E) Nasal cancer
Reasoning:
1. Analyze the main symptoms...
...
Conclusion: E) Nasal cancer

[Additional examples follow the same format]
Question: {{question}}
{{answer_options}}
Reasoning:
```

A.4 Chain-of-Thought + Few-Shot

This configuration combines the step-by-step reasoning of CoT with the contextual guidance of Few-Shot examples. The model is first shown example questions with detailed reasoning steps, and then asked to analyze a new question in a similar manner.

```
The following are multiple choice questions (with answers) about medical knowledge.
Each example includes step-by-step reasoning leading to the correct answer.

[Example 1 with reasoning]
Question: A 65-year-old man with a history of diabetes, hypertension, smoking, and
dyslipidemia presents with right nasal obstruction persisting for 6 months,
associated with serosanguineous rhinorrhea. Physical examination reveals no
significant findings. What is the most likely diagnosis?
A) Allergic rhinitis
B) Vasomotor rhinitis
C) Nasal polyps
D) Rhinofibroangioma
E) Nasal cancer
Reasoning:
1. Identify key symptoms and risk factors...
2. Analyze each option...
3. Conclude the correct diagnosis based on reasoning...
Answer: E) Nasal cancer

[Additional examples follow the same pattern]

Now analyze the following question using the same step-by-step reasoning approach:
Question: {{question}}
{{answer_options}}
Reasoning:
```

A.5 Self-Reflection

This configuration implements a two-stage reasoning process. The model first generates an initial answer with associated confidence and reasoning paths, and then reflects on its reasoning, iteratively improving its answer until a certain confidence threshold is reached or other stopping criteria are met.

Stage 1: Initial Analysis Template

```
Act as a medical expert. Evaluate and select the best alternative.
Provide your answer in the following format:

{
    "alternative": "letter of selected option",
    "confidence": "confidence level between 0 and 1",
    "key_issues": ["key issue 1", "key issue 2"],
    "reasoning_path": "detailed explanation of reasoning"
}

Question: {{question}}
{{answer_options}}
Analysis:
```

Stage 2: Reflection Template

```
Evaluate this medical response:
Initial response: {{initial_response}}
Analyze:
1. Is the confidence level justified? (0-1)
2. Identify up to 2 key issues in the reasoning
3. Suggest a concrete improvement in the analysis
Evaluation:
```

The self-reflection process continues iteratively until:

1. A confidence threshold of 0.8 is reached
2. The maximum number of iterations (default: 3) is reached
3. No improvement is detected in consecutive iterations

A.6 MEDPROMPT

This configuration combines dynamic few-shot selection, chain-of-thought reasoning, and choice-shuffling, aiming to improve the robustness and quality of the final answer.

```
1. Stage 1: Dynamic Few-Shot Selection
Select the k most similar questions from the training set using embedding similarity.

2. Stage 2: Chain-of-Thought Generation
For each selected example:
Question: {{example_question}}
{{example_answer_options}}
Generate a step-by-step reasoning leading to the correct answer {{correct_answer}}.

3. Stage 3: Final Template
# Context: The following are medical questions with their answers and detailed
    explanations.
[The k most similar examples are inserted here]

# New Question:
{{question}}
{{answer_options}}

# Instructions:
1. Analyze the previous examples similar to this question
2. Develop step-by-step reasoning
3. Identify key concepts
4. Select the most appropriate answer
5. Provide a detailed explanation
Reasoning:
```

Appendix B Multi-Agent Configuration Description

This appendix provides detailed information about the implementation of multi-agent configurations used in our study of GPT-4o's performance on Spanish-language medical examinations. We developed and validated multiple collaborative reasoning strategies, ranging from basic voting mechanisms to sophisticated multi-agent frameworks.

B.1 Basic Voting Mechanisms

The foundation of our multi-agent approach began with three voting mechanisms: simple majority voting (see Figure B1), weighted voting (see Figure B2), and Borda count (see Figure B3). In simple majority voting, five independent medical expert agents analyzed each question and selected their preferred answer, with the final selection determined by plurality. The weighted voting mechanism enhanced this approach by incorporating confidence levels, where each agent assigned probability weights to all answer options, with weights summing to 1.0. The Borda count method implemented a

ranked-choice system, where agents ranked all options and points were assigned based on ranking positions, allowing for more nuanced preference expression.

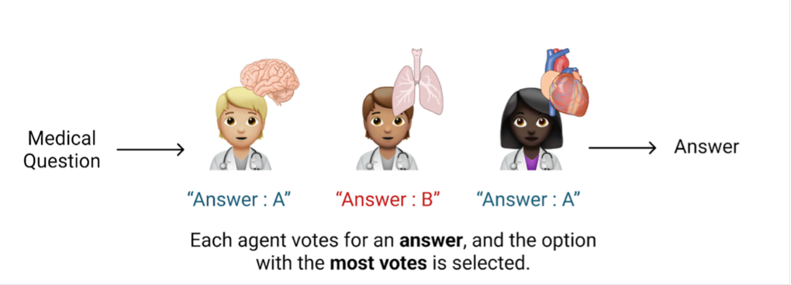

Fig. B1 Simple majority voting mechanism: each agent votes for an answer, and the option with the most votes is selected.

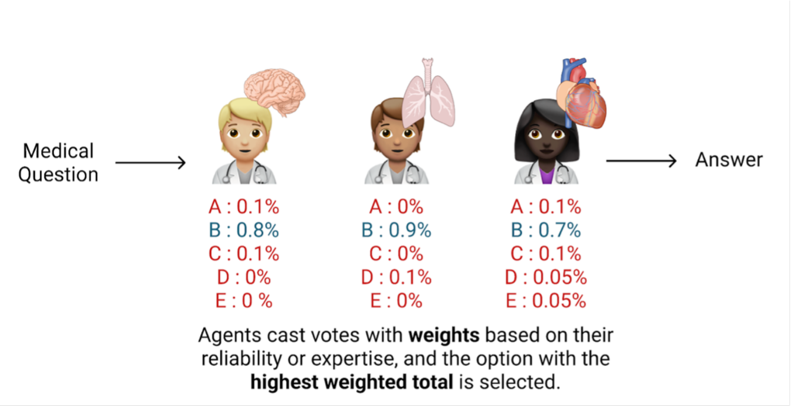

Fig. B2 Weighted voting mechanism: agents assign weights based on confidence levels, and the option with the highest weighted total is selected.

B.2 MEDAGENTS Framework

The MEDAGENTS framework employs a structured five-stage pipeline that emulates multidisciplinary collaboration for medical reasoning (see Figure B4for an overview). This framework is meticulously designed to enhance the decision-making capabilities of GPT-4o by leveraging the interactions of specialized agents, each contributing their domain expertise to solve complex medical queries. The process begins with Expert Gathering, where specialists are recruited based on the content and requirements of the medical question. This ensures that a diverse range of domain expertise—such as cardiology, pediatrics, and other relevant specialties—is included in the problem-solving process. Following this, the Analysis Proposition stage is initiated. In this phase, each

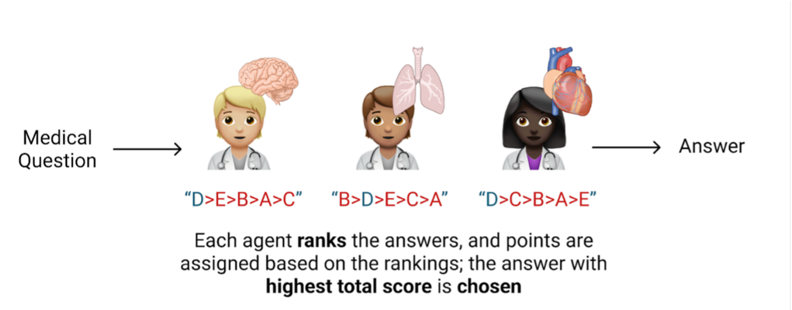

Fig. B3 Borda count mechanism: agents rank all options, and points are assigned based on ranking positions, with the answer having the highest total score chosen.

agent independently conducts a detailed analysis using criteria specific to their specialty. This includes evaluating potential differential diagnoses, interpreting evidence, and applying their knowledge to the question at hand. Once individual analyses are completed, the framework transitions to Report Summarization. Here, a dedicated synthesis agent consolidates the independent analyses into a cohesive report. This report identifies areas of consensus among the specialists and highlights any remaining divergences that require further discussion. The framework then enters the Collaborative Consultation phase, where agents engage in iterative discussions to refine their evaluations. Structured protocols are implemented to facilitate the resolution of disagreements, ensuring that the final outcome is well-informed and supported by robust consensus-building processes. The final stage is Decision Making, where a weighted aggregation of inputs from all specialists is conducted. This process considers the relevance and confidence of each specialist's contribution to produce a definitive answer. The result is a comprehensive and evidence-based conclusion, supported by a detailed rationale that reflects the multidisciplinary expertise involved.

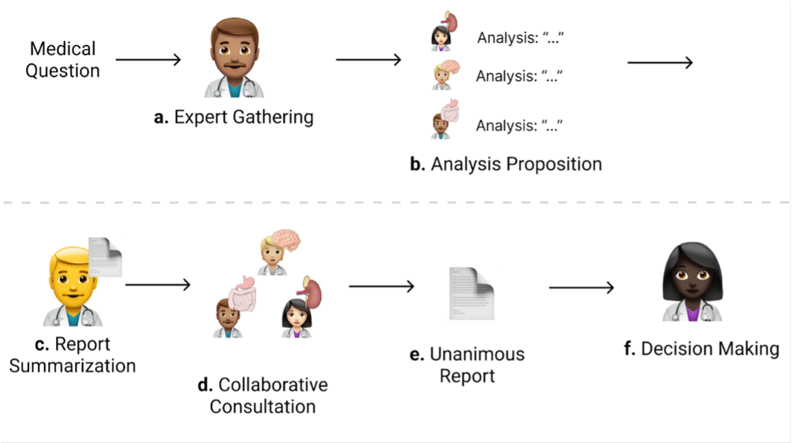

Fig. B4 Overview of the MEDAGENTS framework: a structured pipeline that integrates multidisciplinary expertise for medical reasoning.

B.3 MDAGENTS Framework

The MDAgents framework is a dynamic and adaptive approach to medical decision-making that tailors its complexity and collaboration strategy based on the requirements of the medical query. The process begins with a complexity check, where each medical question is assessed to determine its level of intricacy. This evaluation categorizes questions into three distinct levels: low, moderate, or high complexity. The classification relies on predefined medical criteria, such as the number of medical domains involved, the need for interdisciplinary expertise, and the overall intricacy of the task—ranging from straightforward, fact-based inquiries to complex differential diagnoses. Once the complexity level is identified, the expert recruitment process activates. For low-complexity cases (see Figure B5), a single primary care agent, typically a generalist, is responsible for handling the query. These cases are straightforward and involve minimal diagnostic challenges, allowing for a streamlined approach. On the other hand, moderate-complexity scenarios (see Figure B6) necessitate a Multidisciplinary Team (MDT) composed of 3 to 5 specialists, each contributing their domain-specific expertise. These agents collaborate to address cases requiring the integration of knowledge across multiple medical fields. For the most challenging high-complexity cases (see Figure B7), an Integrated Care Team (ICT) is assembled. This team consists of 5 to 7 specialists who work together in a structured, multi-layered manner to address intricate and multifaceted medical problems that require exhaustive analysis and interaction. The analysis and synthesis process varies depending on the complexity level. For low-complexity questions, the generalist agent employs prompting techniques such as Few-Shot or Chain-of-Thought reasoning to arrive at a direct answer without further refinement. These methods ensure that simple tasks are resolved efficiently, avoiding unnecessary computational resources. For moderate-complexity cases, the MDT engages in a hierarchical collaboration process, where each specialist analyzes the question independently before entering iterative discussions to refine their perspectives. This structured interaction allows the agents to resolve disagreements and reach a consensus over several rounds of dialogue. High-complexity cases follow a more extensive procedure. Here, ICT members conduct individual assessments and produce detailed reports, which are then synthesized through a collaborative discussion process. The result is a comprehensive and refined final answer, moderated to ensure all perspectives and evidence are fully integrated. The final decision-making stage synthesizes all contributions into a well-informed and evidence-based answer. In low-complexity cases, the generalist agent's direct answer is sufficient. For moderate-complexity questions, the MDT collaboratively decides on the outcome after thorough discussion. For high-complexity queries, the ICT generates an exhaustive report that captures all diagnostic and therapeutic considerations, reflecting a robust and multidisciplinary approach.

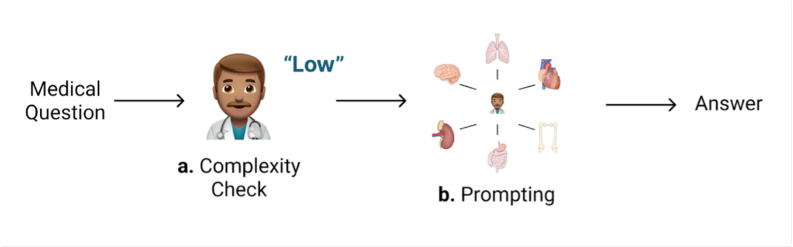

Fig. B5 MDAGENTS framework for low-complexity cases: a single primary care agent employs simple reasoning techniques to provide a direct answer.

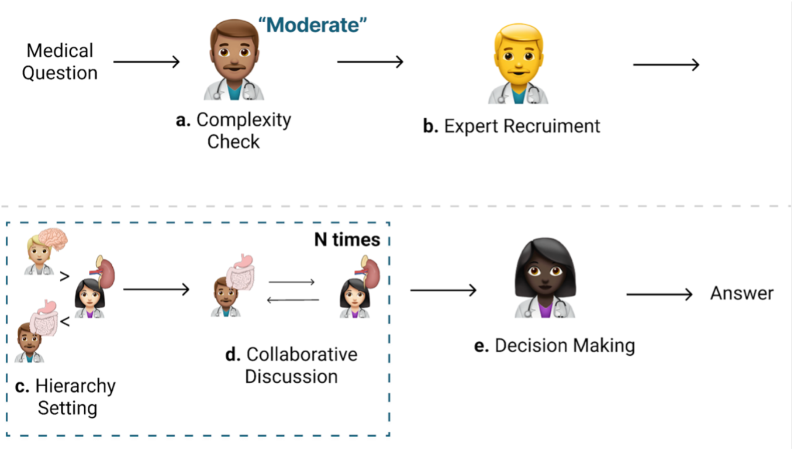

Fig. B6 MDAGENTS framework for moderate-complexity cases: a multidisciplinary team collaborates through iterative discussions to resolve disagreements and reach a consensus.

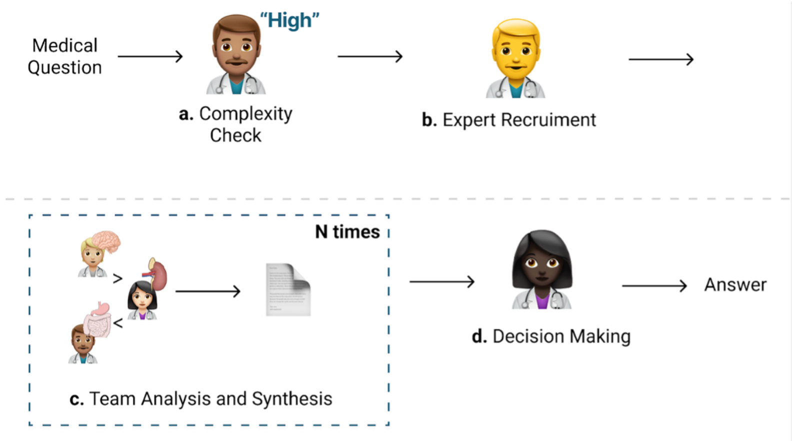

Fig. B7 MDAGENTS framework for high-complexity cases: an integrated care team conducts detailed assessments, produces reports, and synthesizes a comprehensive final answer.

Table C1 Summary of Selected Significant Pairwise Comparisons. Positive mean differences indicate that Model1 outperformed Model2. Adjusted p-values (Benjamini–Hochberg) are shown.

**Comparison**	**Mean Difference**	**Adjusted p**
MDAGENTS vs. ZERO-SHOT	+43.17	< 0.01
MEDAGENTS vs. ZERO-SHOT	+22.17	< 0.01
COT + FEW-SHOT vs. ZERO-SHOT	+18.67	< 0.01
COT vs. ZERO-SHOT	+10.17	< 0.01
MEDPROMPT vs. ZERO-SHOT	+11.17	< 0.01
MDAGENTS vs. SELF-REFLECTION	+48.83	< 0.01
COT + FEW-SHOT vs. SELF-REFLECTION	+24.33	< 0.01
FEW-SHOT vs. SELF-REFLECTION	+16.00	< 0.01
MDAGENTS vs. COT	+33.00	< 0.01
MEDAGENTS vs. COT	+12.00	< 0.01

Table C2 Kruskal–Wallis Tests for the Effect of Temperature (0.3, 0.6, 1.2) on Model Performance. No significant differences were observed (ns = not significant).

**Model**	**Metric**	**N**	**H**	**p-value**	**Significance**
BORDA COUNT	Score	6	1.5909	0.63525	ns
COT	Score	6	1.5441	0.63525	ns
COT + FEW-SHOT	Score	6	2.5000	0.63525	ns
FEW-SHOT	Score	6	3.4286	0.63525	ns
MDAGENTS	Score	6	2.2794	0.63525	ns
MEDAGENTS	Score	6	0.8571	0.79567	ns
MEDPROMPT	Score	6	4.5714	0.63525	ns
SELF-REFLECTION	Score	6	0.2941	0.94930	ns
VOTING	Score	6	1.8382	0.63525	ns
WEIGHTED VOTING	Score	6	0.0758	0.96300	ns
ZERO-SHOT	Score	6	3.7121	0.63525	ns

Appendix C Statistical Analysis Results

C.1 Pairwise Comparisons

C.2 Kruskal–Wallis Tests

Appendix D Supplementary Results by Specialty

This appendix provides a more granular distribution of accuracy outcomes by specialty across all models considered in the investigation. The table below Table D3) displays the number of questions in each specialty classified into four categories of accuracy performance:

- 0% Accuracy: No model answered these questions correctly.
- 1%-50% Accuracy: Between 1% and 50% of the models answered these questions correctly.
- 51%-99% Accuracy: Between 51% and 99% of the models answered these questions correctly.
- 100% Accuracy: All models answered these questions correctly.

This distribution helps in understanding not only the average performance but also the consistency of the models' responses across different medical domains. For instance, a high number of questions with 100% accuracy in a specialty suggests that those question types are well-covered by the model. Conversely, specialties with many questions in the lower accuracy brackets indicate areas where further improvement is needed.

Table D3 Accuracy Distribution by Specialty

Specialty	0% Accuracy	1%-50% Accuracy	51%-99% Accuracy	100% Accuracy
Cardiology	1	4	3	32
Surgery	0	1	3	36
Dermatology	6	6	11	144
Endocrinology	0	1	1	8
Gastroenterology	0	3	6	31
Gynecology	2	3	6	39
Hematology and Oncology	1	2	0	17
Infectious Diseases	2	5	5	37
Nephrology	2	3	3	32
Neonatology	3	6	2	29
Neurology	1	0	3	36
Obstetrics	0	6	4	30
Ophthalmology	10	3	14	51
Otolaryngology	10	10	2	55
Pediatrics	4	2	4	40
Psychiatry	1	2	6	71
Respiratory Medicine	0	8	6	26
Rheumatology	2	1	0	17
Public Health	3	7	11	30
Traumatology	5	3	6	35
Urology	0	2	2	17
Urology and Nephrology	0	6	2	12
